# Setting Eyes on the Retinal Pigment Epithelium

**DOI:** 10.3389/fcell.2018.00145

**Published:** 2018-10-24

**Authors:** Tania Moreno-Marmol, Florencia Cavodeassi, Paola Bovolenta

**Affiliations:** ^1^Centro de Biología Molecular “Severo Ochoa”, Consejo Superior de Investigaciones Científicas, Universidad Autónoma de Madrid, Madrid, Spain; ^2^Centro de Investigación Biomédica en Red de Enfermedades Raras, Instituto de Salud Carlos III, Madrid, Spain; ^3^Institute of Medical and Biomedical Education, University of London, London, United Kingdom

**Keywords:** morphogenesis, eye development, squamous epithelial cell, zebrafish (*Danio rerio*), optic cup

## Abstract

The neural component of the zebrafish eye derives from a small group of cells known as the eye/retinal field. These cells, positioned in the anterior neural plate, rearrange extensively and generate the optic vesicles (OVs). Each vesicle subsequently folds over itself to form the double-layered optic cup, from which the mature eye derives. During this transition, cells of the OV are progressively specified toward three different fates: the retinal pigment epithelium (RPE), the neural retina, and the optic stalk. Recent studies have shown that folding of the zebrafish OV into a cup is in part driven by basal constriction of the cells of the future neural retina. During folding, however, RPE cells undergo an even more dramatic shape conversion that seems to entail the acquisition of unique properties. How these changes occur and whether they contribute to optic cup formation is still poorly understood. Here we will review present knowledge on RPE morphogenesis and discuss potential mechanisms that may explain such transformation using examples taken from embryonic *Drosophila* tissues that undergo similar shape changes. We will also put forward a hypothesis for optic cup folding that considers an active contribution from the RPE.

## Introduction

All the organisms of the animal kingdom that bear a visual sensing organ share the need of protecting the light-receptive cells with a pigmented structure (Martinez-Morales, 2016). In the vertebrate eye, this structure is represented by the retinal pigment epithelium (RPE). The RPE consists of a monolayer of cells positioned at the back of the neural retina. One of its prominent features is the production and accumulation of pigment granules in specialized organelles, the melanosomes. These organelles are responsible for quenching the excess of light that may otherwise damage the photoreceptors (Strauss, 2005), the retinal cells in charge of sensing and processing light input. A second feature of the RPE cells is a highly elaborated apical membrane with extensions that surround and closely interact with the outer segment of the photoreceptors. This organization is essential for other functions that the RPE bears on photoreceptor homeostasis. These include the cyclic and circadian rhythms-dependent phagocytosis of the photoreceptor outer segment; the recycling of water and ions generated during the high photoreceptor metabolic activity; the secretion of growth factors and an active participation in visual phototransduction (Strauss, 2005; Letelier et al., 2017). Thus, RPE-photoreceptor dependence is such that they can be considered as a single functional unit. This is also reflected by the pathological consequence that RPE impairment has on the function and survival of photoreceptors. This occurs, for example, in genetically or environmentally triggered degenerative diseases that lead to partial or total vision loss, such as different forms of Retinitis Pigmentosa (Letelier et al., 2017).

The RPE is a neuroectodermal derivative. In zebrafish, its specification begins in cells that occupy the dorso-medial portion of the optic vesicle (OV), which is the first morphologically recognizable primordium of the eye (Kwan et al., 2012). All OV cells are initially alike and express a small network of regulatory genes – such as the transcription factors Otx2, Pax6, Rx, Six3, Six6, and Lhx2 – essential for the acquisition of eye identity (Fuhrmann, 2010; Beccari et al., 2013). Inductive signals initiate the specification of the targeted OV cells into three derivatives: the RPE, the neural retina and the optic stalk (Fuhrmann, 2010; Fuhrmann et al., 2014). The regulatory mechanisms driving neural retina patterning and its morphogenesis are fairly well known (Martinez-Morales et al., 2017). RPE specification, which entails an important morphological and functional divergence from a “neural” phenotype, is instead less well understood. Similarly, the impact that RPE specification has on eye morphogenesis has been poorly addressed. Here, we will discuss these issues focusing on the RPE of the zebrafish, a species in which RPE cells undergo an extreme transformation from a neuroepithelial to a squamous morphology.

## Current View of Zebrafish Eye Morphogenesis

**FIGURE 1:**
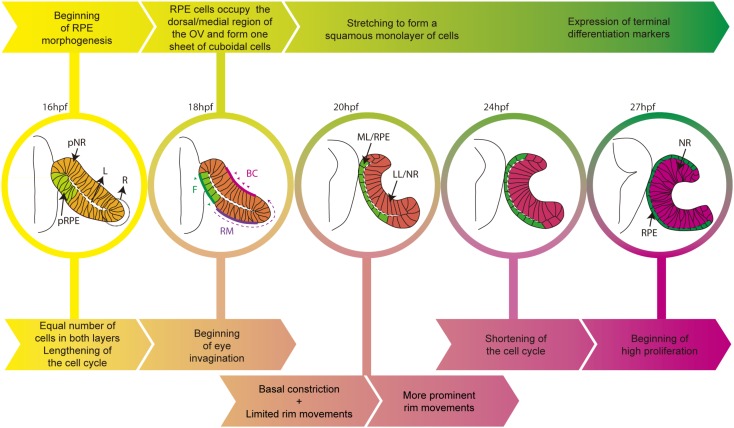
Schematic diagram of the time line of zebrafish eye morphogenesis. The top arrows (yellow to green) indicate the salient events in RPE development whereas bottom arrows (yellow to magenta) those related to the entire eye primordium. Eye structures are color coded: progenitors, light yellow; lateral/neural retina layer, dark pink; medial/RPE layer, green. BC, basal constriction; F, flattening; L, virtual lumen; LL, lateral layer; ML, medial layer; NR, neural retina; pNR, prospective neural retina; R, rim region; RM, rim movements; RPE, retinal pigment epithelium; pRPE, prospective RPE.

The adult zebrafish eye shares strong similarities with that of other vertebrates but its initial morphogenesis occurs with slightly different mechanisms. In amniotes, eye progenitors are bilaterally positioned in the anterior neural plate (Inoue et al., 2000) and protrude to form the OVs as polarized neuroepithelial cells. In the zebrafish instead, progenitors are specified in the center of the anterior neural plate as a single cohesive group of cells (Martinez-Morales and Wittbrodt, 2009; Fuhrmann, 2010; Martinez-Morales et al., 2017). These cells acquire neuroepithelial and polarized characteristics whilst progressively organizing into OVs (Rembold et al., 2006; Ivanovitch et al., 2013). Zebrafish OVs are flat and composed of a folded continuous layer of neuroepithelial cells, so that the apical surface of the two layers, connected by a rim region, face one another separated only by a virtual lumen (Figure 1). This organization differs from the balloon-shaped OV of the chick and mouse embryos. All OV progenitors have the potential to acquire RPE, neural retina or optic stalk identities. However, zebrafish fate map studies have shown that the ventral/lateral layer (abutting the lens ectoderm) originates the neural retina (Li et al., 2000b), whereas the dorsal/medial layer (averting the lens ectoderm) contributes to both the neural retina and the RPE (Figure 1). Indeed, a good proportion of medial layer cells undergo “epithelial flow” around the rim (also called rim involution, Figure 1), emitting dynamic lamellipodia that attach to the extracellular matrix (ECM) and generate the necessary force to rotate into the lateral layer (Li et al., 2000b; Kwan et al., 2012; Heermann et al., 2015; Sidhaye and Norden, 2017). So far, there is no evidence of a similar type of flow during mammalian or avian eye morphogenesis. Nevertheless, in teleost, this event results in the imbalance of cell number between the two layers, possibly contributing to the concomitant modifications of cell shape the two layers undergo. Indeed, cells of the lateral layer undertake basal constriction mediated by actomyosin contractility (Martinez-Morales et al., 2009; Nicolas-Perez et al., 2016). The result is cone-like cells with a reduced basal but enlarged apical surface (Sidhaye and Norden, 2017). This rearrangement, together with apico-basal elongation and lateral compaction of the prospective retinal cells, promotes the inward folding of the OV and the formation of the optic cup (OC; Figure 1; Bogdanović et al., 2012; Heermann et al., 2015; Nicolas-Perez et al., 2016; Sidhaye and Norden, 2017).

As the ventral/lateral layer expands and undergoes shape changes, the remaining cells of the dorsal/medial OV layer begin to acquire RPE identity and transform their appearance (Figure 1). Similar to the other OV cells, these cells are initially organized as a pseudostratified epithelium but soon align their nuclei and reduce their height along their apico-basal axis to form a cuboidal monolayer of cells. In amniotes, this is the final shape of the RPE (Bok, 1993) but, in teleost, cuboidal cells further flatten and originate an array of squamous polygonal cells (Figure 1; Li et al., 2000b). One of the yet unanswered questions is whether this cellular transformation is a “passive” process. That is, RPE cells could change shape and stretch in response to external forces exerted by the expanding apical surface of the neural retina and possibly by other surrounding tissues. This is a possibility given that few medial layer cells (future RPE) need to overlay and match the extension of the apical neural retina surface, in virtual absence of RPE cell proliferation (Cechmanek and McFarlane, 2017). In a different and not mutually exclusive view, the RPE could instead be programmed to stretch cell autonomously, as a consequence of regulated and active rearrangements of its own cytoskeleton and adhesive properties. So far, this possibility has not been directly tested but in the *Drosophila* embryo there are examples of tissues undergoing a similar conversion. The underlying mechanisms have been studied and could provide clues regarding RPE flattening, as we detail below.

## From a Pseudostratified Epithelium to a Squamous Monolayer of Cells

**FIGURE 2:**
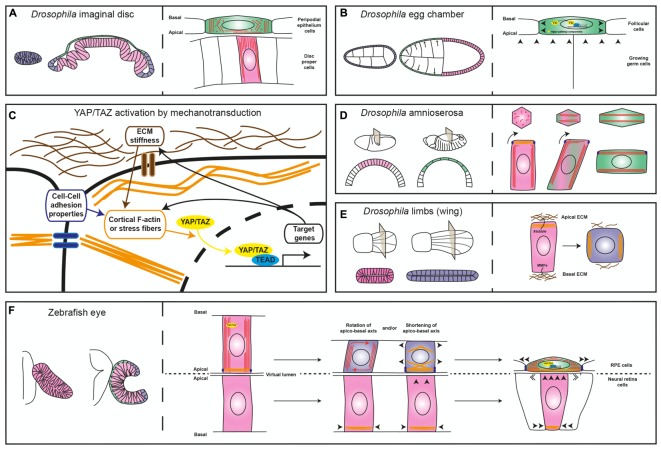
Diagrams comparing different mechanisms of epithelial flattening. **(A)** Left, the drawing depicts the immature *Drosophila* imaginal disk composed of a homogeneous layer of cuboidal cells (blue) and the mature disk composed of a columnar epithelium, the disk proper (pink) and a squamous layer, the peripodial epithelium (green). Right, the enlarged view depicts the differential microtubule (red lines and dots) organization of the two layers. **(B)** The left drawing depicts the development of the *Drosophila* egg chamber, which is surrounded by the follicular cells. The egg chamber is initially composed of a homogeneous cuboidal epithelium (blue) that then becomes flat in the anterior region (green) and columnar in the posterior one (pink). The right drawing depicts the contribution of Yorkie (Yki) to follicular cell flattening. **(C)** Schematic representation of the relation between sources of cell tension and YAP/TAZ cellular location (cytoplasmic vs. nuclear). **(D)** On the left, view of the *Drosophila* embryo at two different stages of development and relative cross sections showing the changes the amnioserosa undergoes (columnar in pink and squamous in green). The right panel depicts epithelial flattening of the amnioserosa cells driven by perpendicular rearrangement of the long axis of the cell (modified from Pope and Harris, 2008). **(E)** Left, view of two developmental stages of the *Drosophila* limb (the wing) and relative cross sections showing cell shape changes from columnar (pink) to cuboidal (blue). The right panel depicts ECM degradation and reorganization of myosin-II, which promote cell shortening. **(F)** Left, schematic representation of the zebrafish optic vesicle and optic cup, with the RPE in green, the neural retina in pink and the rim in blue. The right panel depicts two possible mechanisms that could contribute to RPE flattening, as discussed in the text. In all panels, arrowheads indicate mechanical forces; microtubules are represented in red, acto-myosin filaments in orange, ECM in brown and junctions in violet.

The *Drosophila* imaginal disks are among such examples. These epithelial sac-like structures present in the larva originate most of the adult appendages during metamorphosis (Whittle, 1990). Initially, the entire imaginal disk is composed of cuboidal cells that, through a differential re-arrangement of microtubules (Tang et al., 2016), differentiate into a layer of columnar and elongated cells, or disk proper, and a squamous peripodial epithelium (Figure 2A; McClure and Schubiger, 2005). The acquisition of the peripodial morphology involves Decapentaplegic (Dpp) (McClure and Schubiger, 2005), a member of the TGF-beta family of signaling factors. Suppression of Dpp signaling (McClure and Schubiger, 2005) or RNAi-mediated interference with the peripodial expression of the transcription factor Yorkie/YAP (Fletcher et al., 2018), an effector of the Hippo pathway, is sufficient to prevent stretching of the peripodial membrane, suggesting that this is an active process, regulated at the transcriptional level. Similar results were obtained with Yorkie/YAP manipulation in the *Drosophila* ovarian follicular epithelium (Fletcher et al., 2018), which undergo dramatic flattening during growth of the egg chamber (Kolahi et al., 2009). In these cells, decreased concentration at the apical membrane of upstream Hippo pathway components – i.e., the Crumbs-Expanded and Merlin-Kibra protein complexes – or inactivation of kinase Warts, leads to Yorkie/YAP nuclear localization, which is required to actively promote further cell flattening (Figure 2B; Fletcher et al., 2018).

These results are not surprising given that the transcriptional regulators YAP and the highly related TAZ are emerging as pivotal mediators of the cell self-perception within its environment, so that mechanical inputs from the surrounding promotes YAP/TAZ nuclear localization and, thus, a context-dependent gene expression response (Figure 2C; Totaro et al., 2018). On the other hand, in *yap* medaka fish mutants the entire body lacks tension as also observed in a YAP-deficient human RPE cell line (Porazinski et al., 2015). This is because YAP – but not TAZ that seems to control only cell proliferation in medaka fish – is required for the expression of ARHGAP18, a RhoGAP that suppresses F-actin polymerization. As a consequence, cells have less cortical actomyosin and do not deposit properly fibronectin fibrils in the surrounding ECM, making all tissues prone to collapse (Porazinski et al., 2015). Thus, YAP/TAZ has a double and likely feed-forward role, not necessarily dependent on Hippo pathway activation (Totaro et al., 2018). It acts as a mechano-transducer of the environmental tension by shuttling from the cytoplasm to the nucleus (Figure 2C), as well as a determinant of tissue tension by directly or indirectly controlling the expression of regulators of cytoskeletal dynamics.

In this scenario, YAP/TAZ appear appropriate candidates to mediate RPE cell flattening. However, loss of *Yap* function in mouse is embryonic lethal before the OV begins to form (Morin-Kensicki et al., 2006), whereas conditional inactivation of *Yap* in the optic primordia induces the prospective RPE territory to adopt a neural retina-like identity (Kim et al., 2016). Notably, the latter phenotype is also observed in mouse embryos after genetic inactivation of *Otx1/2*, *Mitf* and β-catenin, genes considered as RPE determinants (Nguyen and Arnheiter, 2000; Martinez-Morales et al., 2001; Westenskow et al., 2009). So far it is unclear if the function of YAP in the mouse RPE is linked to Hippo, or related to Wnt*-*β-catenin signaling, as observed in other contexts (Totaro et al., 2018), or perhaps both or none. Nevertheless, the expression of Otx2 and of two RPE terminal differentiation markers, Sox9 and Ezrin, is missing in the prospective RPE of *Yap* mutants. This indicates that YAP acts rather upstream in the regulatory network controlling RPE specification (Kim et al., 2016) and makes it difficult to determine its possible implication in RPE flattening. A window of opportunity to address this question may come from the zebrafish. In this species, the combined abrogation of *yap* and *taz* alleles lead to an eye that lacks the RPE, although it is still unclear if their progenitors are initially formed and then fail to undergo differentiation or are, for example, incorporated in the neural retina. Embryonic transplanted cells null for *yap* and *taz* do not contribute to RPE formation, whereas forced expression of *yap* in neural retinal progenitors induce the formation of pigment granules, indicating that Yap/Taz act cell-autonomously imposing RPE characteristic. This activity seems to depend on their nuclear interaction with Tead transcription factors (Miesfeld et al., 2015). In contrast, mutants in *yap* alone develop an RPE that, however, is discontinuous (Miesfeld et al., 2015). The patchy RPE appearance can be interpreted as a failure of RPE specification only in a subset of progenitors but it might also reflect poor cell-to-cell adhesion or alterations in cytoskeletal dynamics, as observed in *yap* medaka mutants. Whether the latter interpretation is correct needs further investigation but there is evidence that cytoskeletal dynamics during epithelial morphogenesis may be linked to the same mechanisms that promote fate specification, as observed during the first lineage separation the mammalian morula undergoes. This occurs at the transition from 8 to 16 blastomere stage during which, individual blastomeres acquire the capacity to interpret their relative position with respect to the inside-outside of the embryo (Mihajlovic and Bruce, 2017). Outside blastomeres are specified as trophectoderm (future placenta) and adopt a flat epithelial conformation, whereas inside blastomeres form the inner cell mass (the origin of the future embryo). This lineage bifurcation depends on the differential activation of the Hippo pathway. In prospective trophectodermal cells, the Hippo pathway is inhibited and Yap/Taz accumulates in the nucleus forming Yap/Taz/Tead complexes that activate the expression of lineage specific genes, such as *Cdx2* (Nishioka et al., 2009). At the same time, Yap/Taz lead to the acquisition of a well-defined apico-basal polarity, a differential distribution of adherens junction and cytoskeletal components with respect to the inner cell mass, thereby enabling cell flattening (Mihajlovic and Bruce, 2017). Thus, specification and stretching of both trophectoderm and RPE require Yap/Tead activity, suggesting functional parallelisms.

Besides the possible involvement of YAP/TAZ as drivers of RPE morphogenesis, changes in cytoskeletal organization and modifications of cell-to-cell contacts are additional active mechanisms that could promote RPE cell flattening. These mechanisms have been observed in the conversion of the amnioserosa cells of the gastrulating *Drosophila* embryo from columnar to squamous epithelium (Figure 2D). This conversion occurs thanks to a 90° rotation of their cellular components – including microtubule bundles, centrosome, nucleus and endoplasmic reticulum, which changes the long axis of the cell perpendicular to its initial position. This phenomenon seems to be initiated autonomously by the bending of the growing microtubules that, in the columnar shaped amnioserosa, find resistance to their growth in the apical accumulation of actin filaments. Notably, microtubule rotation is accompanied by remodeling of the adherens junctions dependent on myosin contraction (Figure 2D; Pope and Harris, 2008). At the moment, microtubule dynamics during zebrafish OC formation has been analyzed only in the context of epithelial flow at the rim, without finding a significant contribution to this process (Sidhaye and Norden, 2017). However, thin sectioning of the zebrafish OV shows the possible rotation of the nuclear longitudinal axis in the prospective RPE cells as they undergo flattening (see Figure 1B in Li et al., 2000b). Thus, the amnioserosa flattening model (Pope and Harris, 2008) may be relevant for explaining zebrafish RPE morphogenesis. Modifications may be nevertheless needed as the RPE cells transit through an intermediate cuboidal shape absent during amnioserosa transformation. This intermediate shape may depend on ECM remodeling, as observed in morphogenetic elongation of the epithelium of the *Drosophila* limbs (Figure 2E). In the limbs, the transcriptionally regulated expression of matrix proteases releases the epithelium from apical and basal ECM constraints and allows the re-localization of myosin-II from the apical to the lateral membranes. The result is a columnar to cuboidal transition and a consequent expansion of the tissue surface (Diaz-de-la-Loza et al., 2018). In theory, a similar mechanism could explain how the RPE matches the apical surface of the folding neural retina.

In the above-mentioned examples of epithelial transformations, there seems to be only a marginal or no contribution of cell proliferation and cell death. Both processes are likely dispensable or marginally involved also in RPE differentiation. Cell death is negligible at early steps of zebrafish eye formation and there is an increase in the length of the progenitor cell cycle during the period spanning from OV to OC transition (Li et al., 2000a). Thus, the total number of cells barely changes as the RPE flattens. Furthermore, pharmacological inhibition of cell proliferation causes small but properly patterned eyes (Kwan et al., 2012; Heermann et al., 2015; Cechmanek and McFarlane, 2017; Sidhaye and Norden, 2017).

## A Model of Optic Cup Formation Based on Tissue Collaboration

In the first section, we have indicated that the basal constriction of neural retinal cells and cell flow around the rim act as motors of OC invagination (Bogdanović et al., 2012; Nicolas-Perez et al., 2016; Sidhaye and Norden, 2017). Both processes contribute to a notable expansion of the neural retina apical surface, which may be sufficient to generate pressure on the closely apposed apical surface of the future RPE. This conclusion comes from interference studies performed by bathing the entire embryo with drugs that inhibit cytoskeletal organization (Nicolas-Perez et al., 2016; Sidhaye and Norden, 2017). However, in these conditions the prospective RPE cytoskeleton is also compromised, making it difficult to exclude its possible contribution to the failure of OC folding. Similar consideration applies to the analysis of the interaction of the basal surface of the prospective neural retina cells with laminin, an ECM component deposited at the basal surface of both OC layers (Bryan et al., 2016; Sidhaye and Norden, 2017). Knock-down of laminin subunits reduces the contractility of the basal surface of the retinal layer and perturbs cell translocation at the rim, thereby impairing OV folding (Nicolas-Perez et al., 2016; Sidhaye and Norden, 2017). Whether there are concomitant RPE morphological alterations remains to be carefully analyzed.

With these considerations in mind, folding of the zebrafish OV into a cup could be modeled as a morphogenetic process that requires the contribution of the dorso/medial and ventro/lateral layers. The expansion of the apical retinal layer may be an initial trigger that exerts tension on the overlying layer, thereby forcing YAP nuclearization and the activation of Tead-mediated transcription of tissue determinants (i.e., *Otx* genes) as well as a YAP-related control of cytoskeletal dynamics (Figure 2F). Changes in cytoskeletal dynamics, in turn, may promote prospective RPE flattening either by simply shortening the long axis of the neuroepithelial cell or by favoring the perpendicular rotation of its long axis (Figure 2F). These changes may increase RPE stiffness, which feed-back into the retina, promoting the flow of ventro/medial cells through the rim, as previously proposed (Heermann et al., 2015). The rim flow seems in fact independent from the neural retina basal constriction, as it is still present in the medaka *opo* mutants (Bogdanović et al., 2012), in which a mutation in a transmembrane protein implicated in retinal basal adhesion prevents OC folding (Martinez-Morales et al., 2009). In contrast, expansion of the RPE surface driven by cell stretching and cytoskeletal-mediated increased stiffness may “push” rim cells into the retina (Heermann et al., 2015).

Other studies support an active role of the RPE in OC morphogenesis. The RPE can bend autonomously in culture (Willbold et al., 1996). In eye-organoids derived from mammalian embryonic stem cells (Eiraku et al., 2011; Nakano et al., 2012), significantly high levels of phospho-myosin accumulation make the RPE stiffer than the neural retina as determined by atomic force microscopy (Eiraku et al., 2011). This and other observations lead to a proposal that the RPE forms a rigid shell around a softer retina and this differential tension together with the apical constriction of rim cells induces OC formation (Eiraku et al., 2012). Furthermore, in absence of Wnt signaling, the mouse RPE does not extend and the OV does not fold, suggesting that the RPE has to reach a sufficient size to sustain the curvature of the OC (Carpenter et al., 2015). Besides, in the zebrafish *otx* morphants and *Otx* null mice, in which the RPE does not form, the neural retina is everted (Martinez-Morales et al., 2001; Lane and Lister, 2012), indicating that the RPE is needed for neural retina invagination.

Thus, it seems reasonable to propose that tension and extension of the RPE can be crucial for proper OC folding just as much as basal constriction of the retinal cells or epithelial flow at the rim. This hypothesis needs experimental verification.

## What Comes Next

4D analysis of the embryonic zebrafish has provided a wealth of information on eye morphogenesis, although full comprehension of RPE formation is lagging behind and many questions remain open. For example, there are still uncertainties on how many of the movements sustaining zebrafish eye morphogenesis can be fully applied to the formation of the amniote eye. Meanwhile, details of the gene regulatory network leading to zebrafish RPE specification are unclear. In mice, this network includes Wnt-βcatenin signaling and the activity of the transcription factors Yap, Otx, Mitf followed by Pax6 and Sox9, which both contribute to maintain RPE identity (Nguyen and Arnheiter, 2000; Martinez-Morales et al., 2001; Masuda and Esumi, 2010; Raviv et al., 2014; Kim et al., 2016). In zebrafish, *otx* genes have a similarly essential role in RPE specification but *mitf* genes seem to be dispensable (Lane and Lister, 2012), whereas *yap* inactivation gives a phenotype that differs from that of the mouse, as already discussed. These differences might be explained by the zebrafish genome duplication or the presence of additional related genes that have adopted their function as proposed in the case of *mitf* (Lane and Lister, 2012). Alternatively, they may derive from species specific adaptations of the RPE gene regulatory network. Identification of such adaptations may help to understand if there is any functional significance in the different shape of the vertebrate RPE: cuboidal in amniotes but squamous in teleosts.

## Author Contributions

TM-M, FC, and PB conceived and wrote the manuscript.

## Conflict of Interest Statement

The authors declare that the research was conducted in the absence of any commercial or financial relationships that could be construed as a potential conflict of interest.
